# Eurasian and African mitochondrial DNA influences in the Saudi Arabian population

**DOI:** 10.1186/1471-2148-7-32

**Published:** 2007-03-01

**Authors:** Khaled K Abu-Amero, Ana M González, Jose M Larruga, Thomas M Bosley, Vicente M Cabrera

**Affiliations:** 1 Mitochondrial Research Laboratory, Department of Genetics, King Faisal Specialist Hospital and Research Center, Riyadh, Saudi Arabia; 2 Department of Genetics, Faculty of Biology, University of La Laguna, Tenerife, Canary Islands, Spain; 3 Neurology Division, Cooper University Hospital, Camden, NJ, USA

## Abstract

**Background:**

Genetic studies of the Arabian Peninsula are scarce even though the region was the center of ancient trade routes and empires and may have been the southern corridor for the earliest human migration from Africa to Asia. A total of 120 mtDNA Saudi Arab lineages were analyzed for HVSI/II sequences and for haplogroup confirmatory coding diagnostic positions. A phylogeny of the most abundant haplogroup (preHV)1 (R0a) was constructed based on 13 whole mtDNA genomes.

**Results:**

The Saudi Arabian group showed greatest similarity to other Arabian Peninsula populations (Bedouin from the Negev desert and Yemeni) and to Levantine populations. Nearly all the main western Asia haplogroups were detected in the Saudi sample, including the rare U9 clade. Saudi Arabs had only a minority sub-Saharan Africa component (7%), similar to the specific North-African contribution (5%). In addition, a small Indian influence (3%) was also detected.

**Conclusion:**

The majority of the Saudi-Arab mitochondrial DNA lineages (85%) have a western Asia provenance. Although the still large confidence intervals, the coalescence and phylogeography of (preHV)1 haplogroup (accounting for 18 % of Saudi Arabian lineages) matches a Neolithic expansion in Saudi Arabia.

## Background

This study represents mtDNA data regarding the population of Saudi Arabia. Geographically, desert is the most prominent feature of the Arabian Peninsula, which comprises the modern countries of Saudi Arabia, Yemen, Oman, the United Arab Emirates, Qatar, Bahrain, and Kuwait. Saudi Arabia occupies eighty percent of the Arabian Peninsula and is divided into five major regions – Central, Northern, Southern, Eastern and Western. From the western coastal region (At-Tihamah), the land rises from sea level to a peninsula-long mountain range (jabal al-Hijaz) beyond which are plateaus to the east. The southwestern 'Asir region has mountains as high as 3,000 metres (9,840 ft) and is known for having the most hospitable climate in the country. The east is primarily rocky or sandy lowland continuing to the shores of the Arabian Gulf. Although vast arid tracts dominate, stretches of coastline along the Arabian Gulf and the Red Sea and several major oases in the central and eastern regions have provided water necessary for human habitation. The coastal areas have been trading centers for centuries with resultant population diversity. In addition, for 1400 years the Haj has brought millions of Muslims annually to the region between Mecca and Jeddah, some of whom have stayed for generations. Traditionally, the central (arid) region of the country has had more population stability. More than 95% of the population now is settled in population centers that are mainly located along the eastern and western coasts and near interior oases such as Hofuf, Buraydah, and Riyadh.

The Arabian Peninsula is a region through which numerous migrations between Africa and Asia took place since ancient times. Anthropological [1,2], archaeological [3], and genetic [4,5] evidence has given support to the hypothesis that modern humans may have dispersed out of Africa, following a southern route through the Arabian Peninsula before they pursued a Levantine route [6]. According to this scenario, the Arabian Peninsula may have been the first step in the colonization of southern and eastern Asia. Middle Palaeolithic artefacts discovered in southwestern areas of the Arabian peninsula are similar to ones recovered in Africa, providing support for the suggestion that the Red Sea coasts may have been important in this southern expansion [7]. The presence of obsidian lithics on the African and Arabian sides of the Red Sea attests to Neolithic contacts as well. Archaeological evidence supports late Neolithic Levantine colonization of the Arabian Peninsula with successive population expansions and contractions depending on climatic conditions [8].

The strategic position of the Arabian Peninsula made it a crucial area for trade, cultural exchange, and warfare after the emergence of Old World Western civilizations. Mesopotamian states invaded the Arabian Peninsula from the north since prehistoric times [9], Ionic and Roman-Byzantine classic cultures took control of strategic trade routes in Arabia, and the Sassinid Persians dominated southern Arabia around 575 AD. Influences from the African side were also present as Pharanoiac Egypt and the Sudanese Meroitic and Abyssinian Askumite kingdoms extended their borders well inside Arabia [10]. Arabian Nabatean and Sabean cultures exerted their influence in turn on the Levant and Ethiopia, although to a lesser degree. Events changed dramatically with the rise of Islam in Arabia during the 7^th ^century AD. In a short span of time, Arabs built a military and cultural empire that extended from Pakistan in the east to the Iberian Peninsula in the west. Even more complete Arabization occurred later in North Africa with the Bedouin Hilalian invasion in the 11^th ^century AD.

The impact of these migrations on the Arab gene pool remains unclear because genetic information about the region has been scarce. Arab populations (Bedouin, Saudi, and Yemenite) are distinct from other Near East populations and from India and Central Asia in an analysis based on classical markers, suggesting the possibility of an ancient expansion from East Africa [11]. Early studies could not discriminate remote from recent contacts, but non-recombining uniparental markers have allowed more refined phylogeographic analysis at both continental [12,13] and regional [14,15] levels.

The rapid mutation rate of mitochondrial DNA (mtDNA) and Y-chromosome microsatellites permits estimates of lineage expansion age and of the most probable geographic origin of these expansions [16,17]. Only two studies regarding the Arabian Peninsula have been based on mtDNA. Lineage classification of a small sample of 29 Bedouins [18] revealed that 25 (86%) had a Eurasian origin, two (7%) belonged to the sub-Saharan Africa L0 and L2 haplogroups, and two were left undetermined. A study of 115 Yemeni mtDNAs showed that Eurasian-specific and African-specific lineages existed in almost equal proportion in that southern Arabian Peninsula sample [19].

In a sample of 120 Saudi Arabs, we sequenced the non-coding HVSI/II mtDNA regions and further characterized haplogroup diagnostic coding region positions by restriction fragment length polymorphism (RFLP) or by partial sequencing in order to estimate the genetic structure of the Arabian Peninsula and to search for archaic N and/or M lineages such as those found in India, Australia, and Southern-east Asia that trace a rapid human expansion outside Africa. The comparison of this sample to 2,204 classified sequences from the Near East and 728 from East Africa allowed us to estimate the relative gene flow between these areas and the Arabian Peninsula. We also provide a detailed mtDNA phylogeny of haplogroup (preHV)1, the most frequent and diverse haplogroup in the Arabian Peninsula. The analysis of this haplogroup, recently renamed R0a [20], is based on complete sequences and a global phylogeographic analysis based on 255 HVSI sequences.

## Results

The total number of different haplotypes in our sample of 120 Saudi Arabs were 107 (K = 89%) when HVSI and II variation and RFLP were taken into account [see Additional file 1]; however, the K value dropped to 64% when only partial HVSI variation was used in comparison with other populations (see Table 1). Some lineages had to be included into imprecise groups such as H/HV/R for haplotype and haplogroup frequency comparison, although all Saudi haplotypes were completely sorted into their respective clades and sub-clades [see Additional file 1]. The bulk of individuals (86%) belonged to the Eurasian macrohaplogroup N and its main R branch (75%), while the Sub-Saharan Africa macrohaplogroup L (7%) and the Asian macrohaplogroup M (7%) accounted for a smaller proportion of haplotypes.

**Table 1:** Haplogroup and macrohaplogroup frequencies in the Near East and eastern-African populations^1^, gene diversity (H) with standard error (± s.e.) and percentage of number of haplotypes per sample size (K)

	**Tuk**	**Kur**	**Irn**	**Irq**	**Syr**	**Pal**	**Drz**	**Jor**	**Bed**	**Ara**	**Yem**	**Egy**	**Sud**	**Eth**	**Ken**
	**494**	**212**	**712**	**116**	**119**	**118**	**45**	**145**	**29**	**120**	**214**	**126**	**159**	**344**	**99**
Refs^2^	^3,4,7,12,14,15,16^	^5,14,1516^	^6,13,14,15,16,20^	^1,16^	^18,19,20^	^18,20^	^16^	^20^	^16^	^20^	^9,16,18^	^10,17^	^10,20^	^9,18^	^2^
CRS	0.14	0.11	0.08	0.07	0.06	0.07	0.02	0.10	-	0.03	0.03	0.06	0.01	-	-
H/HV/R	0.09	0.04	0.05	0.07	0.09	0.12	0.02	0.05	0.07	0.02	0.04	0.04	0.04	0.02	-
H/HV/R	0.13	0.09	0.07	0.16	0.11	0.12	0.07	0.16	-	0.08	0.03	0.05	-	-	-
V	0.01	-	0.01	-	0.03	-	-	-	-	-	-	0.01	0.02	-	-
H/HV	0.01	0.01	0.01	0.03	0.02	-	0.02	0.01	-	-	-	-	-	-	-
HV	0.01	-	0.03	0.04	0.01	-	-	0.01	-	-	0.01	-	-	-	-
HV1	0.02	0.02	0.01	-	0.02	0.01	0.09	0.02	-	-	0.01	-	0.01	0.03	-
HV2	-	-	0.02	0.01	-	-	-	0.01	-	-	0.01	-	-	-	-
HV/R	0.01	-	0.01	-	-	-	-	-	-	-	-	-	-	-	-
(preHV)1	0.01	-	0.01	0.04	0.03	0.03	0.04	0.03	0.14	0.18	0.09	0.02	0.04	0.10	0.01
R	-	0.01	0.01	-	-	-	-	-	-	-	-	-	-	-	-
R2	-	0.01	0.01	0.01	0.03	-	-	0.01	-	-	0.01	-	-	-	-
R5	-	-	0.01	0.01	-	-	-	-	-	-	-	-	-	-	-
B	-	0.01	0.01	0.01	-	-	-	-	0.03	-	-	-	-	-	-
F	-	-	-	-	-	0.01	-	-	-	-	-	-	-	-	-
J	0.03	-	0.04	0.04	0.05	0.03	0.04	0.02	-	0.06	0.03	0.03	0.01	0.01	0.01
J1	0.02	0.02	0.04	0.03	0.01	0.02	-	0.01	0.03	0.03	0.04	-	-	-	-
J1a	-	0.01	-	-	0.01	0.01	0.02	0.01	-	-	-	-	-	-	-
J1b	0.01	0.02	0.03	0.04	0.01	0.02	-	0.01	0.14	0.12	0.04	0.02	-	-	-
J1b1	0.01	-	-	-	-	-	-	-	-	-	-	-	-	-	-
J1d	0.02	0.03	0.02	0.02	0.01	0.03	-	-	0.03	0.02	0.01	0.02	0.01	0.01	-
T	0.04	0.08	0.04	0.03	0.04	0.04	-	0.01	-	0.01	-	0.02	0.01	0.01	-
T1	0.03	0.02	0.03	0.04	0.03	0.03	0.04	0.01	0.03	0.01	0.01	0.07	0.01	0.02	-
T2	-	-	0.01	0.01	0.03	0.02	-	0.03	-	-	-	0.02	-	0.01	-
T3	0.01	0.01	0.01	-	0.01	0.02	-	0.02	-	0.03	-	0.02	-	0.01	-
T4	-	-	-	-	-	0.01	-	-	-	-	-	0.02	-	-	-
T5	-	-	-	-	0.01	0.01	-	-	0.03	0.02	-	0.01	-	-	-
U	0.02	-	-	0.02	-	-	-	0.01	-	-	0.01	-	-	-	-
U1	-	-	-	-	-	-	-	-	-	-	-	-	-	-	-
U1a	0.04	0.01	0.02	0.01	0.03	0.01	0.07	0.01	0.07	0.01	0.01	0.01	-	-	-
U1b	0.01	-	-	-	0.03	-	-	0.02	-	0.01	-	-	-	-	-
U2b	-	-	-	-	-	-	-	-	-	-	0.01	-	-	-	-
U2d	-	-	-	-	-	0.01	-	0.01	-	-	-	-	-	0.01	-
U2e	0.01	0.02	0.01	0.02	0.01	0.01	-	0.01	-	0.01	-	-	-	-	-
U3	0.05	0.03	0.03	0.06	0.07	0.01	-	0.16	0.03	0.03	0.01	0.02	0.01	0.01	-
U4	0.01	-	0.01	0.02	0.03	0.02	-	0.01	-	-	-	0.02	-	-	-
U5	-	-	-	-	-	-	-	-	-	-	-	0.01	-	-	-
U5a1	0.01	0.02	0.01	-	0.01	-	-	0.01	-	-	-	-	-	-	-
U5a1a	0.01	0.01	0.02	0.01	0.01	0.01	-	-	-	0.01	0.01	-	-	-	-
U5b	0.01	-	-	-	-	-	-	-	-	-	-	0.01	-	-	-
U6a	-	-	-	0.01	-	0.01	-	-	-	0.01	-	-	-	-	-
U6a1	-	-	-	-	0.03	-	-	-	-	-	-	0.01	-	0.03	0.01
U6b	-	-	-	-	-	-	-	-	0.07	-	-	-	-	-	-
U7	0.01	0.06	0.07	0.03	0.03	0.03	-	-	-	-	-	0.01	-	-	-
U8b	0.01	0.01	0.01	-	-	-	-	0.01	-	0.01	-	-	-	-	-
U9	-	-	-	-	-	-	-	-	-	0.03	-	-	-	-	-
K	0.05	0.11	0.06	0.05	0.06	0.08	0.20	0.03	-	0.06	0.05	0.02	0.01	0.01	-
A	0.01	-	-	-	0.01	-	-	-	0.03	-	-	-	-	-	-
N2a	-	-	0.01	-	-	-	-	0.01	-	-	-	-	-	-	-
N9	-	-	-	-	-	-	-	-	-	-	-	-	-	-	-
Y1	-	-	-	-	-	-	-	-	-	-	-	0.01	-	-	-
N1a	-	-	-	-	-	-	-	-	-	0.04	0.04	0.02	-	0.02	-
N1b	0.01	0.01	0.02	0.02	0.02	0.01	0.02	0.01	0.03	0.03	-	0.05	-	-	-
N1c	-	0.01	-	-	-	0.02	0.02	-	-	0.02	-	-	-	-	-
W	0.02	0.04	0.02	-	0.03	0.02	-	0.01	-	0.01	-	0.01	0.01	0.01	0.01
X	0.03	0.01	0.02	0.02	0.01	0.04	0.27	0.01	0.03	0.02	0.02	0.02	0.01	0.01	-
I	0.01	0.01	0.01	-	-	-	0.02	0.02	-	0.01	-	0.02	-	0.01	-
N	-	-	0.01	-	-	-	-	0.01	-	-	-	-	-	-	-
I/M/L	0.01	0.02	0.02	-	-	-	-	-	-	-	-	0.02	-	0.01	-
N/L/M	0.03	-	0.02	-	-	0.02	-	0.01	0.03	-	0.01	0.02	0.08	0.01	0.03
M	-	-	0.01	0.01	-	-	-	-	-	0.02	0.02	0.02	-	-	-
M1	-	-	-	-	-	-	-	0.01	0.03	0.01	-	0.05	0.03	0.08	0.02
M1a	-	-	-	-	-	0.02	0.02	0.01	0.03	0.03	0.01	0.05	0.04	0.07	0.02
M3	-	-	0.01	-	-	-	-	-	0.03	0.01	0.02	-	-	-	-
Z	-	-	-	-	-	-	-	-	-	-	-	-	-	-	-
C	0.01	0.02	0.01	-	-	-	-	-	-	-	-	-	-	-	-
G	-	-	0.01	-	-	-	-	-	-	-	-	-	-	-	-
M/D	0.02	-	0.01	-	-	-	-	-	-	-	-	0.02	0.01	-	-
D	0.01	-	-	-	-	-	-	-	-	-	-	-	-	-	-
L*	-	-	-	-	-	0.01	-	0.01	-	-	-	0.01	-	-	0.03
L3b	-	-	-	-	-	0.01	-	0.03	-	-	0.01	0.01	0.02	-	0.06
L3d	-	-	-	-	-	0.03	-	0.01	-	0.01	0.04	-	0.01	0.03	-
L3e1	-	-	-	-	-	0.01	-	-	-	-	-	0.01	-	-	0.05
L3e2	-	-	-	-	0.01	0.01	-	-	-	-	-	-	-	-	0.02
L3e3	-	-	-	-	-	0.02	-	-	-	-	0.02	-	-	-	0.03
L3e4	-	-	-	-	0.01	-	-	-	-	-	-	-	0.01	-	-
L3e5	-	-	-	-	-	-	-	-	-	-	0.01	-	-	-	-
L3f	-	-	-	-	0.01	-	-	-	-	0.01	-	-	0.04	0.02	0.05
L3f1	-	-	-	0.02	-	-	-	0.01	-	-	0.02	-	0.03	0.03	0.03
L3h	-	-	-	-	-	0.01	-	-	-	0.02	-	-	0.03	0.01	0.04
L3i	-	-	-	-	-	-	-	-	-	0.01	0.01	-	-	0.01	-
L3i/N2a	-	-	-	-	-	-	-	-	-	-	-	0.02	0.05	-	-
L3w	-	-	-	-	-	-	-	0.01	-	-	-	-	-	0.03	0.01
L3x1	-	-	-	-	-	-	-	-	-	-	-	-	-	0.03	0.01
L3x2	-	-	-	-	-	-	-	0.01	-	-	-	0.02	-	0.01	-
L4a	-	-	-	-	-	-	-	-	-	-	0.01	-	-	0.01	-
L4a1	-	-	-	-	-	-	-	-	-	-	-	-	0.01	0.04	0.02
L4g	-	-	-	-	0.01	0.01	-	-	-	-	-	0.01	0.03	0.03	0.14
L2a/L2c	-	-	-	-	-	-	-	0.01	-	-	0.01	0.02	0.08	0.01	0.03
L2a1	-	-	-	0.04	0.02	0.03	-	0.01	-	-	0.01	0.02	0.07	0.05	0.03
L2a1a	-	-	-	-	-	0.01	-	-	-	0.01	0.03	-	-	-	-
L2a1b3	-	-	-	-	-	0.01	-	-	0.03	-	-	0.01	0.01	0.03	0.02
L2a2	-	-	-	-	-	-	-	-	-	0.01	-	0.01	0.03	0.02	-
L2a3	-	-	-	-	-	-	-	-	-	-	-	-	0.04	-	-
L2b	-	-	-	-	-	-	-	-	-	-	-	0.01	0.01	0.02	-
L2c2	-	-	-	-	-	-	-	0.01	-	0.01	-	-	-	-	-
L2d	-	-	-	-	-	-	-	-	-	-	0.01	-	0.01	-	0.01
L6	-	-	-	-	-	-	-	-	-	-	0.07	-	-	0.01	-
L5a1	-	-	-	-	-	-	-	-	-	-	-	0.02	0.01	0.01	0.01
L5a2	-	-	-	-	-	-	-	-	-	-	-	0.02	0.03	0.01	0.01
L5b	-	-	-	-	-	-	-	-	-	-	-	-	-	0.01	-
L1b	-	-	-	-	-	-	-	-	-	-	-	0.01	0.01	-	0.01
L1b1	-	-	-	-	0.01	-	-	0.01	-	-	-	-	0.03	0.03	0.01
L1c	-	-	-	0.02	-	-	-	-	-	-	0.01	-	0.01	-	0.02
L1e	-	-	-	-	-	-	-	-	-	-	-	-	-	-	0.01
L0a	-	-	-	-	-	-	-	-	-	-	-	-	0.01	0.01	0.02
L0a1	-	-	-	-	0.01	0.01	-	0.01	0.03	-	0.02	0.05	0.09	0.04	0.05
L0a1a	-	-	-	-	-	-	-	0.01	-	-	-	0.01	0.01	-	0.03
L0a2	-	-	-	0.01	-	-	-	-	-	-	0.04	-	0.01	0.01	0.05
L0f	-	-	-	-	-	-	-	-	-	-	-	-	0.01	-	0.08
L0k	-	-	-	-	-	-	-	-	-	-	0.01	-	-	-	-
L	0.01	0.01	-	0.09	0.07	0.14	-	0.12	0.07	0.07	0.37	0.22	0.65	0.51	0.89
M	0.04	0.04	0.05	0.01	-	0.02	0.02	0.03	0.10	0.07	0.06	0.13	0.08	0.15	0.04
N	0.09	0.11	0.11	0.03	0.06	0.08	0.33	0.07	0.10	0.12	0.07	0.11	0.01	0.05	0.01
R	0.82	0.82	0.80	0.87	0.87	0.74	0.64	0.77	0.69	0.75	0.49	0.48	0.18	0.27	0.03
others	0.03	0.02	0.04	-	-	0.02	-	0.01	0.03	-	0.01	0.05	0.08	0.01	0.03
H	0.979	0.984	0.990	0.992	0.995	0.995	0.946	0.978	0.995	0.986	0.989	0.993	0.989	0.993	0.995
± s.e.	0.004	0.004	0.002	0.004	0.003	0.003	0.019	0.006	0.011	0.004	0.002	0.003	0.003	0.001	0.002
81	56	67	80	53	64	93	67	56	86	83	80	60	65	63	K (%)

^1 ^Population abbreviations: *Tuk *Turkey, *Kur *Kurds, *Irn *Iran, *Irq *Iraq, *Syr *Syria, *Pal *Palestine, *Drz *Druze, *Jor *Jordanian, *Bed *Bedouin, *Ara *Saudi Arabs, *Yem *Yemen, *Egy *Egypt, *Sud *Sudan, *Eth *Ethiopia, *Ken *Kenia.

^2 ^Data source references are detailed in [see Additional file 2].

### Sub-Saharan African macrohaplogroup L lineages

Five of the eight Saudi Arabian L lineages belonged to different L3 sub-clusters. Although L3d is a widespread African clade, the single Saudi representative (Individual 49; [see Additional file 1]) had exact duplicates only in Yemen and Ethiopia [19]. L3f was the most frequent L3 cluster in Yemen and Ethiopia, and the sole Saudi L3f sequence (457) matched an Ethiopian sequence [19]. Sequence 429 was peculiar because it belonged to the recently defined East Africa haplogroup L3i [19] yet lacked the 16223 transition and included the 16318T transversion. The remaining two L3 sequences (221, 430) had L3h designation. One of them (221) harboured 16192–16218 transitions and presented the 16129-16223-16256A-16311-16362 HVSI motif that was first reported in West Africa [21]. The other (430) belonged to the subset of L3h sequences found in Ethiopia [19] and in Tanzania [22] that had the combined 16179–16274 HVSI motif. This haplogroup was present in moderate frequency in Ethiopians and Yemenis [19] but no matches existed between them and the Saudi population. The three remaining Saudi L haplotypes fell into the L2 macrohaplogroup. One of the sequences (433) belonged to the western L2c clade and had matches in West Africa Guineans [21] and in Mozambique [23]. The last two L2 Saudi sequences (225, 452) fell into the widespread L2a cluster [24] and had matches in East Africa and Yemen.

In general the sub-Saharan Africa maternal gene flow to Saudi Arabia was moderate (7%) and fell into the range found for other Arab populations in the Near East [25]. A small portion of this sub-Saharan Africa genetic input could be due to contacts with Yemeni communities from southern Arabia, but the most characteristic Yemeni L6 clade [19] was not present in the Saudi sample.

### Macrohaplogroup M

Five of the eight M Saudi Arab lineages clustered into the M1 African haplogroup [26]. Three of them had the 16359 transition that was diagnostic of the M1a East African cluster, and the remaining one belonged to the rare but widespread M1b1 cluster characterized in the HVSI region by 16185 transition and the 16190d deletion that had been identified in the northwest Africa, Jordan, and the Iberian Peninsula [27]. The other three M sequences belonged to Indian clades. One had the basic motif (16126, 16223) of the M3 haplogroup [28]. A second had the 15928 and 16304 transitions that defined haplogroup M25 [29], although this sequence [see Additional file 1] did not match any of the definite or putative M25 sequences found in India [29-31] or Pakistan [26].

**Figure 1:**
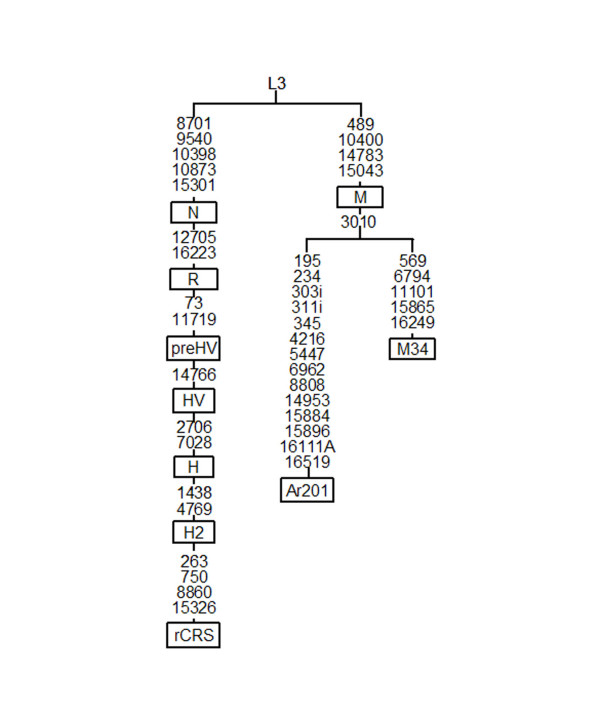
**Phylogenetic position of the haplogroup M Arab 201 sequence**. All mutation differences are listed with respect to the revised Cambridge Reference Sequence (rCRS) [66]. This sequence has accession number DQ904234 in GenBank.

The last M sequence (16111A, 16223) has been found with the central motif in Bhoksa from Uttar Pradesh [32] and with the central motif and the 16129 transition in two derivatives in Yerava from South India [33]. Because these lineages were pooled as undetermined M*, we completely sequenced our sample (Ar201) and compared it to 91 complete Indian M sequences [34-36] to know its phylogenetic position. Our Ar201 sequence shared only transition 3010 with the basal mutations that defined haplogroup M34 [35] so that the most parsimonious tree clustered it with this haplogroup (Figure 1). However, we think that Ar201 may be representative of a new Indian branch of macrohaplogroup M because 3010 is a highly recurrent mutation that has independently appeared in the tips (M40) and sub-cluster roots (D4) of other M haplogroups. The M contributions to the Saudi Arab gene pool represented gene flow from East and North Africa (4%) and India (3%) but not from Central Asia.

### Macrohaplogroup N

All the main western Eurasian branches of N (R, N1a, N1b, N1c, I, W, X) were present in Saudi Arabia, with the least common ones (N1a, N1b, N1c, I, W, X) having an infrequent presence in Saudi Arabs (Table 1). N1a was the only one of these haplogroups that seemed to have a consistent presence across the Arabian Peninsula because it was also moderately frequent (6.9%) and diverse (h = 0.89) in Yemeni [19]. N1a frequency dropped to 4% in Saudi Arabs, where it harboured only two different haplotypes. The most abundant one, with the 16147A-16172-16218-16223-16248-16261-16274-16355 HVSI motif and the 41-73-199-204 HVSII motif, had not been observed in the Near East or in East Africa, and the second (16147G-16172-16223-16248-16355) was only shared with Ethiopians.

Saudi Arabs had the main European and western Asian haplogroups (H, J, T, K, U) included in R, the main branch of N, albeit in different frequencies. Haplogroup H was the most frequent cluster in European (45%) and Near East (25%) populations [16] but only accounted for 13% of Saudi lineages, comparable to the frequency in Bedouin and Yemeni. H frequencies significantly diminished with latitude from Turkey to Yemen through the Levant (r = 0.953; two-tail p < 0.01).

Haplogroups K (6%) and T (7%) had similar frequencies in Saudi Arabs to those found in Europe and the Near East [16]. However, the subgroup composition of haplogroup U clearly differed from Europe in Saudi Arabia and in other Near Eastern regions. The most prevalent haplogroup in Europe (U5) was represented in Saudi Arabs by only one U5a1a derived lineage [see Additional file 1]. Likewise, the North-African U6 haplogroup [15] is represented by only one lineage (1%). Several minority European U sub-clades (U1, U2e, U3, U4, and U7) may have had their origins in the Near East [16]. All of them had representative lineages in Saudi Arabs except for U4, U7, which were also absent from Bedouin of the Negev desert, and Yemeni samples (Table 1).

The rare haplogroup U9 was present in our sample with a frequency of 3% (Table 1). This haplogroup was first defined by RFLP-6383 HaeIII and observed only in South Pakistan [26]. It was later proven to be a sister branch of haplogroup U4 [37] on the basis of two complete U9 sequences (one Ethiopian and one Pakistani), both of which shared the 499–5999 motif. In addition to 6386, transitions at 3531, 3834, and 14094 defined the basal motif of U9. The Ethiopian sequence was considered representative of sub-cluster U9a and the Pakistani sequence as representative of sub-cluster U9b. The three Saudi U9 sequences belonged to U9a because all of them shared the HVSI 16051–16278 motif with the Ethiopian sequence while none of them shared any HVSI or HVSII mutations with the U9b Pakistani sequence ([see Additional file 1]; [37]). These three U9a sequences may be different occurrences of an old implantation of this haplogroup in the Arabian Peninsula.

A feature that differentiated Near Eastern populations from European and West Asian populations was the high frequency of haplogroups J and (preHV)1 [16,38], and this was also true for Saudi Arabia. J haplotypes represented 25% of the Saudi sample, and its main contributor was the J1b cluster (12%). Saudi and Bedouin samples showed an identical trend in this respect and were different from Yemenis, whose J1b frequency (4%) was similar to other Near Eastern samples (Table 1). The J1b frequency in the Arabian Peninsula was significantly higher than in the rest of the Near East, even when Yemenis were included (p < 0.0001). However, J1b in Arabia displayed a low level of haplotypic diversity in spite of its relative abundance (h = 0.57). Unlike the derived J1b1 lineage, J1b was scarce in North Africa [39] and practically absent in Europe [39] except for Italy [40].

Haplogroup (preHV)1 was even more frequent than J1b in Saudi Arabs (18%). The frequency of this sequence in Saudi Arabs was not significantly different from that observed in Yemeni Jews (20.4%) and Bedouins of the Negev desert (14%), but it dropped to 3.4% in Yemenis [19] (Table 1). Like J1b, the (preHV)1 frequency in the Arabian Peninsula was significantly higher than in the rest of the Near East (p < 0.001).

### Phylogeny of haplogroup (preHV)1 based on complete mtDNA sequences

**Figure 2:**
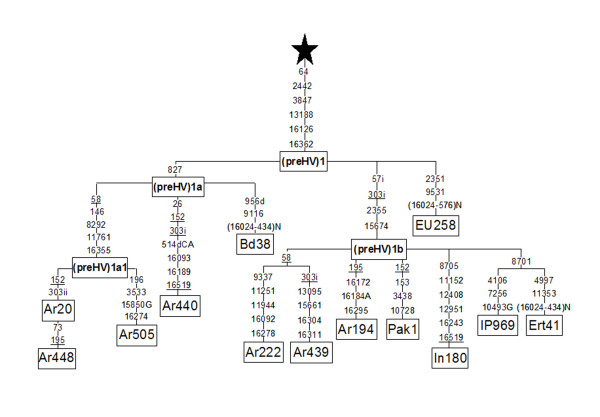
**Haplogroup (preHV)1 phylogeny based on thirteen complete or nearly complete sequences**. The Iberian Peninsula (IP969) and the seven Saudi Arab (Ar) sequences are from this study. Five additional sequences were taken from the literature as detailed in Methods. Numbers along links refer to nucleotide positions with **i **indicating insertions, **d **indicating deletions, underlining indicating recurrent mutations in the (preHV)1 haplogroup. From the star all individuals present the following mutations with respect to rCRS: 263, 750, 1438, 2706, 4769, 7028, 8860, 14766, 15326. All mutation differences are detailed with respect to the revised Cambridge Reference sequence (rCRS) [66]. Our eight sequences were given GenBank accession numbers [GenBank: DQ904235], [GenBank: DQ904236], [GenBank: DQ904237], [GenBank: DQ904238], [GenBank: DQ904239], [GenBank: DQ904240], [GenBank: DQ904241] and [GenBank: DQ904242] for sequences # Ar20, Ar440, Ar505, Ar439, Ar448, Ar194, Ar222 and IP969 respectively.

The relative abundance and diversity of (preHV)1 in the Saudi sample permitted more detailed phylogenetic and phylogeographical analyses of this haplogroup. The phylogenetic tree based on 13 complete mtDNA (preHV)1 sequences (Figure 2) confirmed that the basic motif of this group harboured the 2442, 3847, 13188, 16126 and 16362 transitions [41]. In addition, the transition at 64 was a basic diagnostic mutation for this haplogroup. Three main branches sprouted from this trunk. (preHV)1a was characterized by a transition at 827, while (preHV)1b was defined by the 57i-2355-15674 motif. The 15674 transition was already documented as a defining mutation of a group of (preHV)1 sequences comprising one Druze, one Eritrean, four Ethiopian Jews, and two Yemeni Jews [42]. (preHV)1c was a potential third branch that can be diagnosed by the 9531 transition because this mutation was shared by the EU258 sequence (Figure 2) and a partial sequence from a Moroccan Jew [42]. The Saudi Arab sequences 20, 448, and 505 (Figure 2) constitute a (preHV)1a1 sub-branch within (preHV)1a, defined by transitions 8292, 11761 and 16355. We excluded 58 and 146 as diagnostic positions because 146 was a highly mutable site and because the 58 change was recurrent within (preHV)1 (Figure 2). A (preHV)1b1 sub-cluster was defined by sequences IP969 and Ert41 that shared the 8701 transition. We estimated a radiation age of 18,959 ± 8,478 years for the entire (preHV)1 haplogroup. The (preHV)1a branch, with an age of 9,248 ± 7,604 years, is somewhat younger than the (preHV)1b branch (13,205 ± 7,193).

### Phylogeography of haplogroup (preHV)1

**Figure 3:**
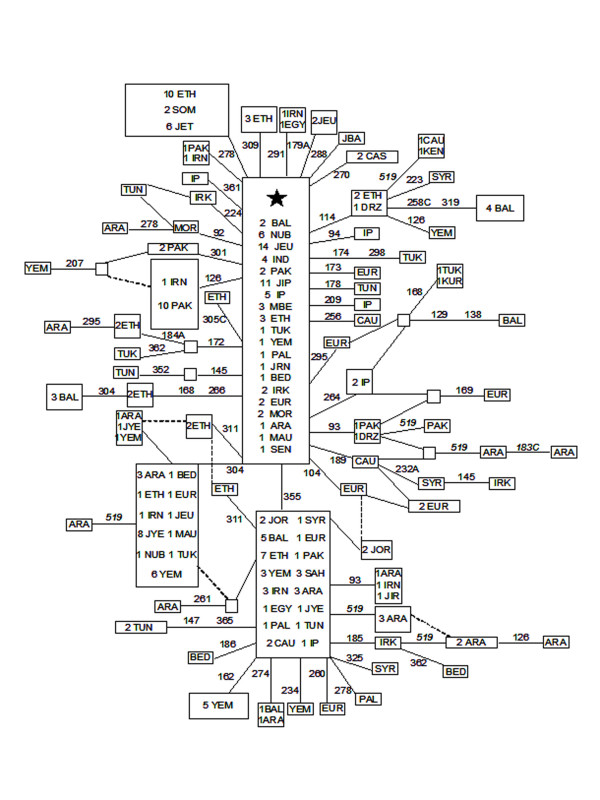
**Reduced median network relating (preHV)1 HVSI sequences**. The central motif (star) differs from rCRS at positions 16126 and 16362 in HVI control region. Numbers along links refer to nucleotide positions minus 16000. Positions not used in diversity estimations are in italics. The broken lines are less probable links and/or recurrent mutations. Size of boxes is proportional to the number of individuals included. Codes are: ARA, Arab; BAL, Balkanian; BED, Bedouin; CAS, Caspian; CAU, Caucasus; DRZ, Druze; EGY, Egyptian; ETH, Ethiopian; EUR, European; IND, Indian; IP, Iberian Peninsula; IRK, Iraki; IRN, Iranian; JBA, Baltic Jew; JET, Ethiopian Jew; JEU, European Jew; JIP, Iberian Jew; JIR, Iraki Jew; JOR, Jordanian; JRN, Iranian Jew; JYE, Yemeni Jew; KEN, Kenian; KUR, Kurd; MAU, Mauritanian; MBE, Moroccan Berber; MOR, Moroccan; NUB, Nubian; PAK, Pakistani; PAL, Palestinian; SAH, Saharan; SEN, Senegalese; SOM, Somalian; SYR, Syrian; TUK, Turkish; TUN, Tunisian; and YEM, Yemeni.

Figure 3 shows the reduced median network obtained from 255 (preHV)1 haplotypes found in a global search comprising nearly 40,000 HVSI sequences. The basic central motif (16126–16362) was the most abundant and widespread, being present in all of northern Africa and in Eurasia from India to the Iberian Peninsula. However, Saudi Arabs were represented by only a single haplotype. The next most abundant clade, defined by 16355 and encompassing the majority of (preHV)1a1 sequences (Figure 2), was overwhelmingly composed of Near East and North African haplotypes with some European outsiders. Saudi Arabs again occupied more peripheral than central positions. The third most abundant clade was characterized by the 16304 transition and probably constituted a sub-cluster of the (preHV)1b branch represented in the genomic tree by the Ar439 sequence (Figure 2). The Arabian Peninsula was the major contributor to this clade.

In addition, several minority clusters provided valuable information. For instance, the one defined by 16309 was formed exclusively by East African sequences. The one identified by 16126 loss or by 16301 was centrally composed of Pakistani and Iranian sequences and had a derivative Yemeni sequence which pointed to some maternal gene flow to Yemen from those areas. The same could be said of the 16172 branch, although the gene flow was from Ethiopia to Saudi Arabia in this case. Ethiopia seemed to have been a secondary center of (preHV)1 expansions to the Near East, Arabian Peninsula, and northwest Africa, as could be deduced from branches defined by 16114 and the motif 16168–16266. Given the peripheral position of Saudi haplotypes, Saudi Arabia seemed to have acted more as receiver than a focus of (preHV)1 expansions with the exception of the 16304 clade. Radiation ages for the whole (preHV)1 haplogroup based on HVSI sequences were 18,993 ± 6,999 years; 9,624 ± 2,994 years for the 16355 ((preHV)1a1) sub-clade, and more recent for the 16304 subclade.

### Population comparisons

We first performed AMOVA using haplogroup and haplotypic frequencies in order to assess the degree of homogeneity within and between the different geographic areas. As customary, the bulk of the variation was found within populations (99.32% for haplotypes and 97.71% for haplogroups). Variance distribution for haplogroups was greater among groups than among populations (1.34% vs. 0.95%), while variance distribution for haplotypes was less among groups than among populations (0.24% vs. 0.44%). Differences were highly significant in all cases (p < 0.001).

**Figure 4:**
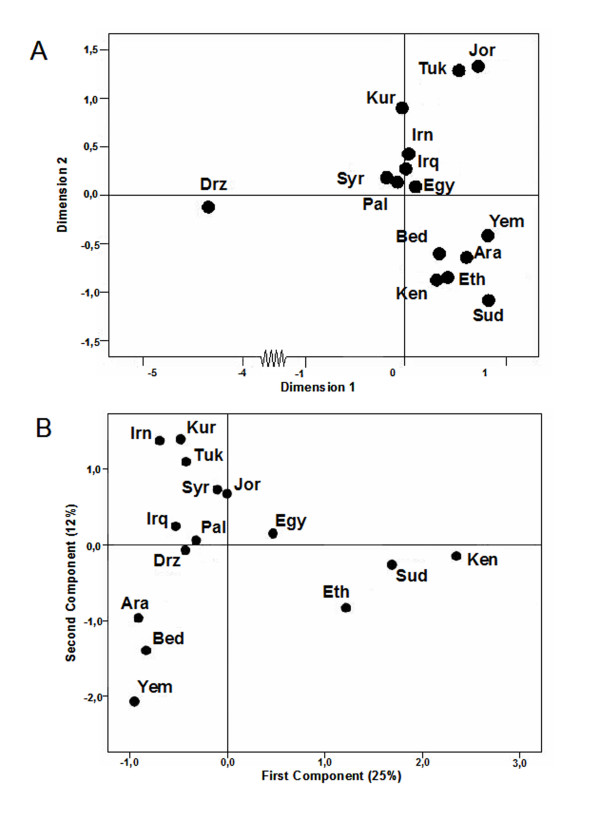
**Graphical relationships among the studied populations**. Codes are as in Table 1. (A) MDS plot based on F_ST _haplotypic distances. Stress value is 0.086. Dimension 1 axe has been shortened to include the Druze sample. (B) PC analysis based on haplogroup frequencies. The two components represent 37% of the total variance.

Pair-wise F_ST _distances based on haplotype frequencies [see Additional file 3] showed that comparatively high heterogeneity within areas was due to the Druze sample that was significantly different from all the other populations, mainly because of a high frequency of haplotypes (27%) belonging to the minority haplogroup X and to K (20%). The Druze sample was a clear outlier in a graphic representation based on F_ST _distances (Figure 4a), separating from the remaining populations along the first dimension. Founder effects or sample bias were the most likely causes of this deviation, as only two X1 and X2 haplotypes [43] accounted for the X percentage. In addition, Druze had the lowest diversity indices of all studied populations (Table 1). The second dimension of this haplotype analysis included the Arabian samples with those of east Africa, while Egyptians were aligned in the cluster of Near East populations.

A somewhat different picture appeared after PC analysis based on haplogroup frequencies (Figure 4b). In this graph, the Druze were not outliers, most probably due to the fact that its variation is not correlated with that in other populations and therefore not reflected by the two first components. The first component separated all the Near East populations from a cluster including Egyptians and other east African groups. The majority of L haplogroups, pulling positively, and haplogroup H, pulling negatively, were predominantly responsible for this split. The second component divided the Near East cluster into three groups. The first comprised northeastern populations characterized by higher frequencies of H haplogroups and absence of L haplogroups. The second combined the Levantine population with Egypt, and the three Arabian Peninsula samples were left in a third group. The major determinants of the Arabian Peninsula singularity were the comparatively high frequency of (preHV)1, J1b, T5 and M3 haplogroups and the population specificity for other haplogroups such as L4, L6, U9 or U6b. This result was similar to that obtained using classical markers [11]. Saudi and Bedouin samples were relatively homogenous; however, the Arabian Peninsula as a whole was not homogenous because Yemenis were differentiated by a greater African component.

## Discussion

MtDNA genetic analysis of this Saudi Arabian group revealed almost exclusively contributions from Africa and the Near East. All Saudi L, M and N lineages were derived from clades with roots in Africa and west and south Asia. The L4, L5, and L6 haplogroups recently found in Ethiopia and/or Yemen [19] were not detected in the Saudi population. Half of the sub-Saharan African Saudi lineages had exact matches in Ethiopians and/or Yemeni, pointing to these areas as the most likely source. The other half belonged to haplogroups with an East Africa origin or that reached the Red Sea in their eastern radiation [19,24]. The Arab slave trade and the expansion of empires from the Sudan and Ethiopia [25] could explain this moderate sub-Saharan Africa maternal contribution to the present Saudi Arabian gene pool.

The majority of M1 lineages in Saudi Arabia belonged to the eastern Africa M1a sub-clade that is particularly frequent and diverse in Ethiopia [19,44]. Ethiopia was again the most likely source. However, the sole M1b1 Saudi sequence probably reached the Arabian Peninsula from northwest Africa through the Levantine corridor because this sequence has been reported repeatedly in west Africa, the Iberian Peninsula, and Jordan [27], but not yet in Ethiopia. Based on Y-chromosome studies, this northern route was proposed as an important path for bidirectional human migration between north Africa and the Levant [45,46]. The remaining M lineages detected in Saudi Arabs had a clear Indian provenance. The basic Saudi M3 lineage out of India was shared by Yemenis and Iranians. Relatively recent contacts between India and the Arabian Peninsula by continental routes through Iran or by Indian Ocean maritime routes could be responsible of this Indian gene flow.

The overwhelming majority of N lineages present in Saudi Arabia had a clear western Asia provenance. Giving priority to geographically closest neighbors, 47% of the N lineages in Saudi Arabs were shared with other Arabian Peninsula neighbors (Bedouin from the Negev and Yemeni), 31% with Levantine populations, 16% with the Anatolian-Caucasus region, and only 6% with eastern Africa. These data revealed only a modest backflow of Eurasian lineages from Africa to the Arabian Peninsula. The close affinity found among Arabian Peninsula populations was due mainly to sharing Eurasian haplotypes and to similar Eurasian haplogroup frequencies and not to the sub-Saharan African contribution that is prominent in the Yemeni population.

The high frequency of (preHV)1 in Saudi Arabians was not significantly different from that found in Bedouin [18] and in Yemeni Jews (20%). However, this (preHV)1 frequency is significantly different of the non-Jewish Yemeni population [19] and may reflect strong genetic drift in the founding population of Yemeni Jews. The frequencies of L (10%) and J (26%) lineages deduced from published sequences of Yemeni Jews [47] were also similar to Bedouin from Negev desert and Saudi frequencies. In general, Jewish communities have evidenced strong maternal founder effects [47,48]. However, they usually harbor chromosome Y and mtDNA lineages that permit their most probable origin to be traced to the Near East because they share the most common haplotypes with those populations [47-50].

The majority of western Asia lineages found in the Arabian Peninsula had original Paleolithic and Neolithic expansions in the Near East [16] or in Caucasian and Caspian regions [26,51]. Most probably, these expansions reached the Arabian Peninsula as secondary waves when climatic conditions there or cultural improvements such as herding allowed colonization. The Arabian Peninsula has had a relatively low population density, and substantial demographic backflow to the Near East is improbable. However, as for M1, minor N North-African influences have been detected by the presence of an U6 lineage in our Saudi sample. It has been suggested that the rare U9 clade might be another interesting exception because it has been detected only in Pakistan [26], Ethiopia, and Yemen [19], and now in our Saudi sample. U9 occurs frequently only among the Makrani population in Pakistan, which is characterized by a large component of sub-Saharan African lineages, suggesting that U9 lineages in Pakistan might also have an African origin [19]. Makrani sub-Saharan Africa lineages have exact matches in Africa, which is compatible with a recent conection as the result of the East African slave trade [26]. However, the entire sequenced Ethiopian and Pakistani U9 lineages [37] are separated by a mean of 4.5 coding mutations from the common root, placing the split at Paleolithic times. Most probably, Ethiopia received its U9 lineages from the Arabian Peninsula that, in turn, received them from northern areas. The southern geographic distribution of U9 contrasts with the west-northern distribution U4, of its sister clade [52], but this is a pattern shared with other Paleolithic U radiations such as U2, U7 [32], or U8 [53] that have eastern and western branches. An original area west to India and east to the Capsian sea would be an equidistant point to conciliate these early U radiations [54].

It is difficult to differentiate successive gene flows or expansions at a population level because the most recent migration could carry both early and derivative lineages. However, the refined phylogenetic and phylogeographic analysis carried out for haplogroup (preHV)1 allows some inferences regarding Arabian Peninsula population history. The coalescence age for the entire (preHV)1 haplogroup was estimated at around 19,000 years ago, which is coincident with the beginning of the last ice age recession. However, in light of the peripheral distribution of the Arabian lineages in the phylogenetic tree (Figure 3), Arabian Peninsula populations most likely did not actively participate in this Paleolithic expansion. The subsequent radiation of the (preHV)1a1 clade occurred around 10,000 years ago, a date that marks the transition from Mesolithic to Neolithic in the Near East. The ancestral core of this cluster was defined mainly by Near Eastern lineages with important Arabian and Ethiopian participation. Finally, a third detectable expansion involving lineages carrying the 16304 transition seemed to be largely restricted to the Arabian Peninsula. Its coalescence age, most probably placed it in a period of empires flourishing in northern Arabia and on both shores of the Red Sea. The lack of archaic N and/or M autochthonous lineages in the Arabian Peninsula do not offer support for the proposed southern route of *Homo sapiens sapiens *outside Africa. Nevertheless, these ancient lineages may become apparent in larger samples.

## Conclusion

The majority of Saudi-Arab mitochondrial DNA lineages (85%) have a western Asia provenance. All of the main western Asia haplogroups were detected in the Saudi sample, including the rare U9 clade. The African contribution totalled 12%, with the sub-Saharan Africa (7%) contribution, represented by L macrohaplogroup, being only slightly higher than the M1 and U6 specific North-African contribution (5%). A small Indian influence (3%) was also detected; however, no archaic N and/or M autochthonous lineages in the Arabian Peninsula were found. Although the still large confidence intervals, the coalescence and phylogeography of (preHV)1 haplogroup (accounting for 18 % of Saudi Arabian lineages) matches a Neolithic expansion in Saudi Arabia.

## Methods

### Study population

Buccal swabs or peripheral blood were obtained from 120 maternally unrelated Saudi Arabs, all whose known ancestors were of Saudi Arabian origin. All five major regions were represented, although the central region was the best represented [see Additional file 1]. Sequence analysis was performed of mtDNA regulatory region hypervariable segment I (HVSI) and hypervariable segment II (HVSII) and of haplogroup diagnostic mutations using RFLPs or partial sequencing when individual haplogroup assignment remained ambiguous. Positions analyzed for each individual are detailed in [see Additional file 1]. For population and phylogeographic comparison, we used 2,204 published or unpublished partial sequences from the Near East and 728 from East Africa, as detailed in Table 1 and [see Additional file 2]. In addition, complete mtDNA sequences were obtained from nine subjects, eight belonging to haplogroup (preHV)1 and one to macrohaplogroup M. Informed consent was obtained from all individuals.

### MtDNA sequencing

Total DNA was isolated from buccal and blood samples using the PURGENE DNA isolation kit from Gentra Systems (Minneapolis, USA). HVSI and HVSII segments were PCR amplified using primer pairs L15996/H16401 and L16340/H408, respectively, as previously described [54]. Complete mtDNA genomes and segments including diagnostic positions were amplified using a set of 24 separate PCRs and single-set cycling conditions as detailed elsewhere [55]. Successfully amplified products were sequenced for both complementary strands using the DYEnamic™ ET dye terminator kit (Amersham Biosciences), and samples were run on MegaBACE 1000 (Amersham Biosciences) according to the manufacturer protocol.

### Haplotype classification

Classification into sub-haplogroups was performed as described previously for African [19,24] and for Eurasian [29, 40, 56] sequences. Published sequences used for comparative genetic analysis were re-classified into sub-clades using the same criteria in order to permit comparison.

### Genetic analysis

Haplotypic diversity was calculated as h [57] and as K (haplotype number/sample size quotient). Only HVSI positions from 16069 to 16365 were used for genetic comparisons of partial sequences with other published data. Genetic variation was apportioned within and among geographic areas using AMOVA by means of ARLEQUIN2 [58]. Four regions were considered: Arabian Peninsula (including Bedouin, Yemeni, and Saudi Arabian samples), Eastern Africa (including samples from Egypt, Sudan, Ethiopia, and Kenya), Levant (containing samples from Jordan, Palestine, Druze, Syria, and Iraq) and Anatolia-Zagros (comprising samples from Turkey, Iran, and Kurds). Pairwise F_ST _distances between populations were calculated from haplogroup and haplotype frequencies, and their significance assessed by a nonparametric permutation test (ARLEQUIN2). Principal component (PC) and multidimensional scaling (MDS) plots were obtained with SPSS version 13.0 (SPSS Inc., Chicago, Illinois). Phylogenetic relationships among partial and complete mtDNA sequences were established using the reduced median network algorithm [59]. In addition to our nine complete sequences, five published complete or nearly complete sequences were used to establish (preHV)1 phylogeny: one European (EU258) [13]; one Pakistani (Pak1) [60]; one Indian (In180) [41], one Bedouin (Bd38) [61], and one Eritrean (Ert41) [61]. A previously compiled database of published and unpublished Eurasian and African sequences [53] was augmented with additional sequences [26,29] and used for (preHV)1 phylogeography.

### Time estimates

Only substitutions in the coding region were taken into account for complete sequences, excluding insertions and deletions. The mean number of substitutions per site compared to the most common ancestor (ρ) of each clade was calculated [62] and converted into time using a substitution rates of 1.26 × 10^-8 ^[63]. For HVSI, the age of clusters or expansions was calculated as the mean divergence (ρ) from inferred ancestral sequence types [62] and converted into time by assuming that one transition within np 16090–16365 corresponds to 20,180 years [64]. The standard deviation of the ρ estimator was calculated as previously described [65}.

## Authors' contributions

KKA was in charge of carrying out the sequences, collecting samples, haplogrouping, and writing part of the manuscript. AMG, JML and VMC were in charge of haplogrouping, designing the experiments, and writing the manuscript. TMB was in charge of writing the manuscript and commenting on historic matters related to this region. All authors read and approved the final manuscript.

## Supplementary Material

Additional File 1 Haplotypes of 120 Saudi Arabs Click here for file

Additional File 2 Refernces used in Haplogroup frequencies comparisons Click here for file

Additional File 3 Matrix of Fst haplotypic distances Click here for file
