# Supplementary material for: Eurasian and African mitochondrial DNA influences in the Saudi Arabian population
Source: BMC Evol Biol. 2007 Mar 1;7:32. doi: 10.1186/1471-2148-7-32 (PMC1810519; doi:10.1186/1471-2148-7-32)
Supplement: Additional File 2 — Refernces used in Haplogroup frequencies comparisons [file 1471-2148-7-32-S2.doc]

Source of the data used for the Near East and eastern-African haplogroup frequency comparisons

1.Al-Zahery N, Semino O, Benuzzi G, Magri C, Passarino G, Torroni A, Santachiara-Benerecetti AS. 2003. Y-chromosome and mtDNA polymorphisms in Iraq, a crossroad of the early human dispersal and of post-Neolithic migrations. Mol Phylogenet Evol 28(3):458-472

2.Brandstätter A, Peterson CT, Irwin JA, Mpoke S, Koech DK, Parson W, Parsons TJ. 2004. Mitochondrial DNA control region sequences from Nairobi (Kenya): inferring phylogenetic parameters fro the establishment of a forensic database. Int J legal Med 118:294-306

3.Calafell F, Underhill P, Tolun A, Angelicheva D, Kalaydjeva L. 1996. From Asia to Europe: mitochondrial DNA sequence variability in Bulgarians and Turks. Ann Hum Genet 60:35-49

4.Comas D, Calafell F, Mateu E, Perez-Lezaun A, Bertranpetit J. 1996.Geographic variation in human mitochondrial DNA control region sequence: the population history of Turkey and its relationship to the European populations. Molec Biol Evol 13:1067-1077

5.Comas D, Calafell F, Bendukidze N, Fananas L, Bertranpetit J. 2000. Georgian and kurd mtDNA sequence analysis shows a lack of correlation between languages and female genetic lineages.Am J Phys Anthropol 112(1):5-16

6.Comas D, Plaza S, Wells RS, Yuldaseva N, Lao O, Calafell F, Bertranpetit. 2004. Admixture, migrations, and dispersals in Central Asia: evidence from maternal DNA lineages.
Eur J Hum Genet. Jun;12(6):495-504

7.Di Benedetto G, Erguven A, Stenico M, Castri L, Bertorelle G, Togan I, Barbujani G. 2001. DNA diversity and population admixture in Anatolia. Am J Phys Anthropol. 115(2):144-156

8.Di Rienzo A, Wilson AC. 1991.Branching pattern in the evolutionary tree for human mitochondrial DNA. Proc Nat Acad Sci USA 88:1597-1601

9.Kivisild T, Reidla M, Metspalu E, Rosa A, Brehm A, Pennarun E,  Parik J, Geberhiwot T, Usanga E, Villems R. 2004. Ethiopian Mitochondrial DNA Heritage: Tracking Gene Flow Across and Around the Gate of Tears. Am J Hum Genet 75:752-770

10.Krings M, Halim Salem A, Bauer K, Geisert H, Malek AK, Chaix L, Simon C, Welsby D, Di Rienzo A, Utermann G, Sajantila A, Pääbo S, Stoneking M. 1999. mtDNA analysis of Nile valley populations: A genetic corridor or a barrier to migration? Am J Hum Genet 64(4):1166-1176

11.Macaulay V, Richards M, Hickey E, Vega E, Cruciani F, Guida V, Scozzari R, Bonné-Tamir B, Sykes B, Torroni A. 1999. The emerging tree of West Eurasian mtDNAs: a synthesis of control-region sequences and RFLPs. Am J Hum Genet 64: 232-249

12.Mergen H, Öner R, Öner C. 2004. Mitochondrial DNA sequence variation in the Anatolian Peninsula (Turkey). J Genet 83(1):101-109

13.Mestpalu M, Kivisild T, Metspalu E, Parik J, Hudjashov G, Kaldma K, Serk P, Karmin M, Behar DM, Gilbert MT, Endicott P, Mastana S, Papiha SS, Skorecki K, Torroni A, Villems R. 2004. Most of the extant mtDNA boundaries in south and southwest Asia were likely shaped during the initial settlement of Eurasia by anatomically modern humans.BMC Genet. 2004 31:5:26

14.Nasidze I, Quinque D, Ozturk M, Benndukidze N, Stoneking M. 2005. MtDNA and Y-chromosome variation in the Kurdish groups. Ann Hum Genet 69:401-412

15.Quintana-Murci L, Chaix R, Wells RS, Behar DM, Sayar H, Scozzari R, Rengo C, Al-Zahery N, Semino O, Santachiara-Benerecetti AS, Coppa A, Ayub Q, Mohyuddin A, Tyler-Smith C, Qasim Mehdi S, Torroni A, McElreavey K. 2004. Where west meets east: the complex mtDNA landscape of the southwest and Central Asian corridor. Am J Hum Genet. 74(5):827-845

16. Richards M, Macaulay V, Hickey E, Vega E, Sykes B, Guida V, Rengo C, Sellitto D, Cruciani F, Kivisild T, Villems R, Thomas M, Rychkov S, Rychkov O, Rychkov Y, Golge M, Dimitrov D, Hill E, Bradley D, Romano V, Cali F, Vona G, Demaine A, Papiha S, Triantaphyllidis C, Stefanescu G, Hatina J, Belledi M, Di Rienzo A, Novelletto A, Oppenheim A, Norby S, Al-Zaheri N, Santachiara-Benerecetti S, Scozari R, Torroni A, Bandelt HJ. 2000. Tracing European founder lineages in the Near Eastern mtDNA pool.Am J Hum Genet. 67(5):1251-1276

17. Stevanovitch A, Gilles A, Bouzaid E, Kefi R, Paris F, Gayraud RP, Spadoni JL, El-Chenawi. 2003. Mitochondrial DNA Sequence diversity in a sedentary population from Egypt

Ann Hum Genet 68:23-39

18.Thomas MG, Weale ME, Jones AL, Richards M, Smith A, Redhead N, Torroni A, Scozzari R, Gratrix F, Tarekegn A, Wilson JF, Capelli C, Bradman N, Goldstein DB. 2002. Founding mothers of Jewish communities: geographically separated Jewish groups were independently founded by very few female ancestors. Am J Hum Genet 701411-1420

19.Vernesi C, Di Benedetto G, Caramelli D, Secchieri E, Simoni L, Katti E, Malaspina P, Novelletto A, Wiel Marin VT, Barbujani G. 2001. Genetic characterization of the body attributed to the evangelist Luke. Proc Natl Acad Sci U S A. 98(23):13460–13463

20. Our umpublished data
